# Dr. John Wade

**Published:** 1912-08-24

**Authors:** 


### Obituary.

DR. JOHN WADE, for some years demonstrator in chemistry at Guy's Hospital, died last week at Sevenoaks Cottage Hospital as the result of a motor-cycle accident. Dr. Wade, whose abilities as a chemist were well known, was born in 1864 at Deptford. He studied at London University and graduated D.Sc. in 1902. His publications were fairly numerous, and perhaps the best known is "Introduction to Organic Chemistry," which has been translated into foreign languages. He frequently contributed papers to the Transactions of the Chemical Society, including the "Boiling-Point of Chloroform," and in 1911 "Apparatus for Constant Pressure in Distillation." He also wrote a report for the Local Government Board in 1907 on "The Destruction of Rats and Ship Disinfection."

